# New Succinimide–Thiazolidinedione Hybrids as Multitarget Antidiabetic Agents: Design, Synthesis, Bioevaluation, and Molecular Modelling Studies

**DOI:** 10.3390/molecules28031207

**Published:** 2023-01-26

**Authors:** Mohammed A. Huneif, Mater H. Mahnashi, Muhammad Saeed Jan, Muhammad Shah, Sultan A. Almedhesh, Seham M. Alqahtani, Mohammad Jamaan Alzahrani, Muhammad Ayaz, Farhat Ullah, Umer Rashid, Abdul Sadiq

**Affiliations:** 1 Pediatric Department, Medical College, Najran University, Najran 55461, Saudi Arabia; 2 Department of Pharmaceutical Chemistry, College of Pharmacy, Najran University, Najran 55461, Saudi Arabia; 3 Department of Pharmacy, Bacha Khan University, Charsadda 24461, Pakistan; 4 Department of Chemistry, COMSATS University Islamabad, Abbottabad Campus, Abbottabad 22060, Pakistan; 5 Department of Pharmacy, Faculty of Biological Sciences, University of Malakand, Chakdara 18000, Pakistan

**Keywords:** succinimide, thiazolidinedione, antidiabetic, α-glucosidase, α-amylase, PTP1B, DPP4 and molecular docking

## Abstract

Diabetes mellitus (DM) is a metabolic disorder majorly arising from the pathophysiology of the pancreas manifested as a decline in the insulin production or the tissue’s resistance to the insulin. In this research, we have rationally designed and synthesized new succinimide–thiazolidinedione hybrids for the management of DM. In a multistep reaction, we were able to synthesize five new derivatives (**10a–e**). All the compounds were new containing a different substitution pattern on the N-atom of the succinimide ring. Initially, all the compounds were tested against the in vitro α-glucosidase, α-amylase, PTP1B, and DPP4 targets. In all of these targets, the compound **10d** was observed to be the most potential antidiabetic agent. Based on this, the antidiabetic activity of the compound **10d** was further investigated in experimental animals, which overall gave us encouraging results. The molecular docking studies of the compound **10d** was also performed against the target enzymes α-glucosidase, α-amylase, PTP1B, and DPP4 using MOE. Overall, we observed that we have explored a new class of compounds as potential antidiabetic agents.

## 1. Introduction

Diabetes mellitus (DM) is a chronic, multifactorial, and progressive syndrome with a disordered metabolism and an inappropriate glycemic condition marked by elevated glucose levels in the blood [1]. Diabetes mellitus is becoming more common throughout the world as a result of unhealthy behaviors and choices; by 2030, the prevalence of DM cases is expected to reach 578 million [2]. Diabetes mellitus can cause atherosclerotic cardiovascular, peripheral arterial, cerebrovascular, and kidney failure, as well as peripheral neuropathy and foot ulcers [3]. The long-term consequences of chronic hyperglycemia include nerve damage, cardiovascular disease, chronic kidney failure (CKF), and many more [4]. Diabetes is typically categorized into two types: type 1 diabetes and type 2 diabetes. T1DM, or insulin-dependent diabetes, is a type of diabetes that is caused by a lack of insulin is one of the most common metabolic disorders in children [5]. T2DM, on the other hand, is still the most frequent type of diabetes, responsible for 90–95 percent of cases [6]. An insulin resistance and a reduced insulin production are at the foundation of this kind of diabetes [7]. Being overweight, a lack of physical activity, and a vitamin D deficiency are all the main risk factors for the initiation and progression of T2DM [8].

The cure for hyperglycemia requires lowering the hepatic glucose synthesis, improving the insulin action, increasing the release of insulin from pancreatic cells, or inhibiting sugar-digesting enzymes [9]. The Food and Drug Administration (FDA) has approved more than ten types of medicines to treat hyperglycemia caused by T2DM [10]. Presently, commercially available oral antidiabetic entities for type 2 diabetes are biguanides, sulfonylureas, thiazolidinediones, meglitinides, and α-glucosidase inhibitors [11,12]. Every class has its own unique approach to controlling the blood sugar levels. One of the pharmacological methods to diabetes prevention is to inhibit the activity of enzymes, including α-glucosidase and α-amylase, which participate in carbohydrate digestion, the glucose nutrient uptake, and the management of postprandial hyperglycemia [13].

**Figure 1:**
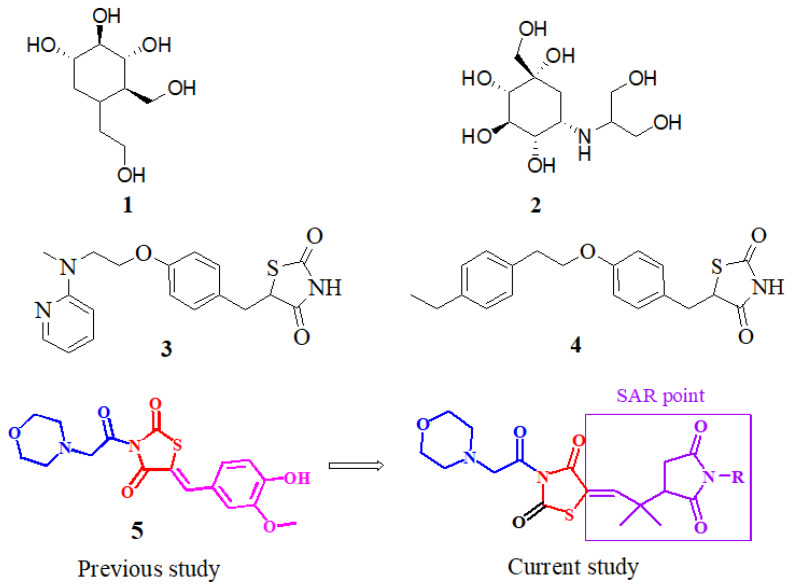
Miglitol (**1**), voglibose (**2**), rosiglitazone (**3**), pioglitazone (**4**), previously reported compound (**5**), and our currently designed compounds.

The α-glucopyranoside link in oligosaccharides is broken down to the release of easily absorbed monosaccharides, glucosidase, and a carbohydrate gastrointestinal enzyme which determine the degree of postprandial hyperglycemia and hence contribute considerably to the beginning and development of T2DM [14]. Alpha-glucosidase inhibitors (AGIs) are oral anti-hyperglycemic medicines that work by inhibiting gastrointestinal alpha-glucosidase in a competing and repeatable manner [15]. As a result, inhibiting these enzymes reduces the pace of glucose absorption into the systemic circulation, making it a viable strategy for combating postprandial hyperglycemia. Miglitol (iminosugar) (1), acarbose, and voglibose (carbasugars) (2) (Figure 1) are α-glucosidase inhibitors that are used to treat late diabetes issues induced by prolonged hyperglycemia. Several state officials have approved the use of these three AGIs for T2DM treatment [16]. The AGIs are used solo or as part of a therapeutic approach. When used alone, the hemoglobin A1c (HbA1c) levels are reduced by 0.8 percent on average [17].

It was previously believed that a single molecule should be used for a single biological target to avoid many of the unwanted effects [18]. However, this conventional approach increases the burden of drug molecules and its metabolites on the body [19]. The unwanted effects are temporary while the metabolites of some drugs can be for a long time within the body and are harmful [20]. Furthermore, the one drug–one target approach is outdated. Organic and medicinal chemists are attempting to develop new drug molecules for more than one purpose [21,22]. The multi-target approach can be for one or more than one disease. Given the knowledge of in silico protein targets [23,24], pharmacophore should be suitably designed to have the capability to show binding interactions with more than one pharmacological target.

The thiazolidine-2,-4-dione (TZD) class of compounds has been shown to regulate high blood glucose levels. Moreover, because of their role and activity in the regulation of several biological pathways, TZD has been the topic of more thorough investigations [25] in terms of its medicinal qualities, such as anti-inflammatory [26], antimicrobial [27], anticancer [28], antihyperglycemic [25], antimalarial, and anti-tubercular (TB) activities [29]. TZDs, such as ciglitazone, rosiglitazone (3), troglitazone, and pioglitazone (4), are insulin-sensitizing drugs beneficial in the diagnosis of T2DM. Aside from these, various researchers have demonstrated the increased glucosidase inhibiting potency of TZDs, or imino-thiazolidinones with an arylidene scaffold. As a result, to produce more potent α-glucosidase inhibitors, the TZD core has been chosen as a desirable heterocyclic motif [30]. During the last few years, it has also been revealed that TZD derivatives are one of the multi-target ligands that can help prevent diabetes by blocking enzymes, including α-glucosidase, α-amylase, and PTP1B. In our previous study, we reported the thiazolidine–vanillin hybrid compound **5** (Figure 1) as a multitarget inhibitor of DPP4, PTP-1B, α-glucosidase, and α-amylase [31]. With an aim to synthesize multipotent antidiabetic compounds, our current research is based on the replacement of vanillin to a pyrrolidine-based core and the morpholine moiety remains unchanged (Figure 1).

## 2. Results

### 2.1. Chemistry of the Synthesized Compounds

**Scheme 1:**
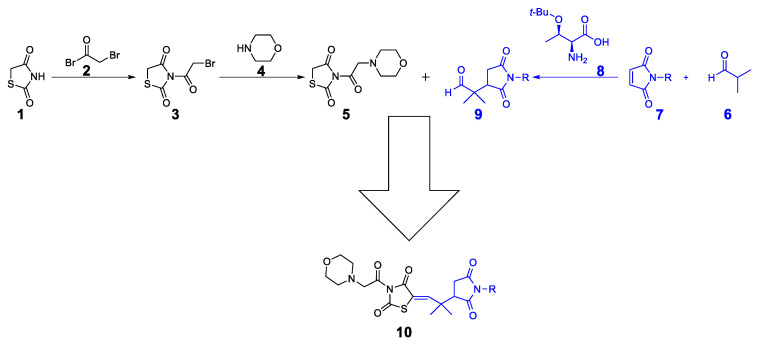
Synthetic approach to final compound **10**.

The synthetic approach for the target compounds’ synthesis is shown in Scheme 1. Initially, the thiazolidine-2,4-dione **1** was protected with bromoacetyl bromide **2** to obtain bromoacetyl-thiazolidinedione **3**. The compound **3** was then protected with morpholine **4** to obtain the target thiazolidinedione hybrid **5**. On the other hand, the Michael product **9** was synthesized by reacting isobutyraldehyde **6** with maleimide **7** (maleimide for **10a**, *N*-methylmaleimide for **10b**, *N*-phenylmaleimide for **10c**, *N*-benzylmaleimide for **10d,** and *N*-4-OMephenylmaleimide for **10e**). As a final step, the Michael product **9** was reacted with morpholine–thiazolidine hybrid **5** to produce the final compounds **10a**–**e**.

**Figure 2:**
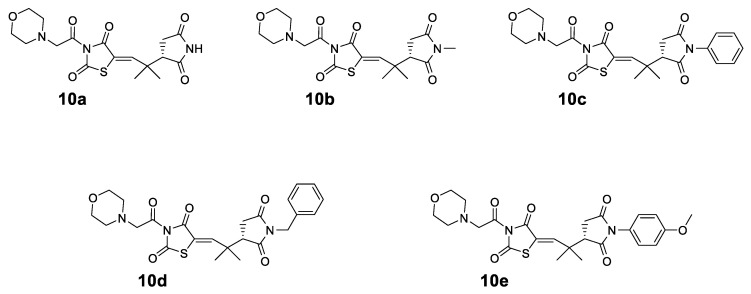
Structures of the synthesized compounds **10a**–**e**.

The structures of the synthesized compounds **10a–e** are shown in Figure 2. In all the synthesized compounds **10a–e**, the common nucleus is a morpholine connected with acetyl-thiazolidinedione and a quaternary dimethyl group to N-substituted succinimides.

The ^1^H NMR (400 MHz in chloroform-D) of compound **10a** was 1.17 (s, 3H), 1.21 (s, 3H), 2.47 (dd, *J* = 16.53, 5.42 Hz, 1H), 2.62 (t, *J* = 6.98 Hz, 4H), 2.87 (dd, *J* = 16.49, 8.79 Hz, 1H), 3.09 (dd, *J* = 5.44, 8.82 Hz, 1H), 3.22 (s, 2H), 3.57 (t, *J* = 7.11 Hz, 4H), 7.86 (s, 1H), and 8.72 (bs, 1H). Similarly, the ^13^C NMR on the same instrument gave chemical shift values of 16.4, 18.2, 29.5, 32.4, 47.6, 53.4, 58.3, 64.5, 117.2, 165.8, 167.6, 172.2, 173.6, 176.1, and 177.8. The analysis calculated for C_17_H_21_N_3_O_6_S C, 51.64; H, 5.35; and N, 10.63 found C, 51.71; H, 5.34; and N, 10.61.

The ^1^H NMR (400 MHz in chloroform-D) of compound **10b** was 1.18 (s, 3H), 1.23 (s, 3H), 2.46 (dd, *J* = 17.21, 5.86 Hz, 1H), 2.61 (t, *J* = 7.11 Hz, 4H), 2.91 (dd, *J* = 17.18, 9.20 Hz, 1H), 2.98 (s, 3H), 3.09 (dd, *J* = 5.85, 9.20 Hz, 1H), 3.25 (s, 2H), 3.60 (t, *J* = 7.04 Hz, 4H), and 7.84 (s, 1H). Similarly, the ^13^C NMR on the same instrument obtained chemical shift values of 17.3, 19.7, 25.6, 28.4, 30.7, 49.0, 52.9, 57.4, 63.4, 116.7, 165.4, 166.9, 170.7, 174.8, 176.3, and 177.4. The analysis calculated for C_18_H_23_N_3_O_6_S C, 52.80; H, 5.66; and N, 10.26 found C, 52.87; H, 5.67; and N, 10.23.

The ^1^H NMR (400 MHz in chloroform-D) of compound **10c** was 1.17 (s, 3H), 1.24 (s, 3H), 2.49 (dd, *J* = 17.30, 5.90 Hz, 1H), 2.59 (t, *J* = 7.14 Hz, 4H), 2.93 (dd, *J* = 17.28, 9.14 Hz, 1H), 3.06 (dd, *J* = 5.89, 9.12 Hz, 1H), 3.24 (s, 2H), 3.61 (t, *J* = 7.14 Hz, 4H), 7.26–7.31 (m, 2H), 7.35–7.41 (m, 1H), 7.42–7.48 (m, 2H), and 7.85 (s, 1H). Similarly, the ^13^C NMR on the same instrument presented chemical shift values of 17.6, 20.1, 25.6, 28.0, 29.3, 49.4, 54.1, 56.7, 64.3, 118.0, 124.2, 128.3, 129.0, 129.3, 134.6, 166.0, 166.7, 169.1, 173.7, 175.8, and 178.7. The analysis calculated for C_23_H_25_N_3_O_6_S C, 58.59; H, 5.34; and N, 8.91 found C, 58.71; H, 5.32; and N, 8.87.

The ^1^H NMR (400 MHz in chloroform-D) of compound **10d** was 1.17 (s, 3H), 1.18 (s, 3H), 2.51 (dd, *J* = 18.40, 5.10 Hz, 1H), 2.63 (t, *J* = 7.10 Hz, 4H), 2.94 (dd, *J* = 18.41, 9.35 Hz, 1H), 3.04 (dd, *J* = 5.10, 9.36 Hz, 1H), 3.27 (s, 2H), 3.62 (t, *J* = 7.09 Hz, 4H), 4.62 (d, *J* = 7.80 Hz, 2H), and 7.23–40 (m, 5H) 7.81 (s, 1H). Similarly, the ^13^C NMR on the same instrument presented chemical shift values of 16.4, 17.2, 25.1, 28.4, 28.9, 41.8, 48.2, 52.7, 56.0, 63.7, 117.5, 125.8, 129.1, 129.8, 132.3, 165.1, 166.4, 168.3, 173.4, 175.0, and 177.4. The analysis calculated for C_24_H_27_N_3_O_6_S; C, 59.37; H, 5.60; and N, 8.65 found C, 59.25; H, 5.62; and N, 8.68.

The ^1^H NMR (400 MHz in chloroform-D) of compound **10e** was 1.22 (s, 3H), 1.25 (s, 3H), 2.54 (dd, *J* = 18.60, 5.50 Hz, 1H), 2.61 (t, *J* = 7.07 Hz, 4H), 2.91 (dd, *J* = 18.60, 9.60 Hz, 1H), 3.08 (dd, *J* = 5.50, 9.61 Hz, 1H), 3.24 (s, 2H), 3.66 (t, *J* = 7.05 Hz, 4H), 3.84 (s, 3H), 6.94 (d, *J* = 7.65 Hz, 2H), 7.11 (d, *J* = 7.65 Hz, 2H), and 7.85 (s, 1H). Similarly, the ^13^C NMR on the same instrument presented chemical shift values of 17.2, 18.2, 24.2, 27.9, 28.4, 48.3, 50.2, 53.8, 56.2, 63.3, 117.0, 126.8, 129.3, 129.8, 134.2, 165.1, 165.9, 169.9, 174.7, 177.2, and 179.7.

### 2.2. In Vitro Antidiabetic Results

The in vitro antidiabetic activities (α-glucosidase and α-amylase) of compounds **10a–e** are summarized in Table 1 in comparison to the standard acarbose. The efficiencies of the compounds were tested on five different serial dilutions with a maximum of 500 and a minimum of 31.25 µM. At the maximum concentration, all the five compounds **10a–e** exhibited very promising results, i.e., 79.49, 79.48, 81.53, 88.43, and 86.44% inhibitions for compounds **10a**, **10b**, **10c**, **10d,** and **10e**, respectively. The observed IC_50_ values for all the compounds were compared with the standard drug acarbose, which presented us with encouraging outcomes. Among all of our compounds, **10d** exhibited the potent alpha glucose inhibitory result with an IC_50_ value of 10 µM in comparison to the acarbose IC_50_ of 6.80 µM. Similarly, in the alpha-amylase enzyme’s results, the compound **10e** was comparatively potent with the IC_50_ value of 9.66 µM in comparison to the standard (IC_50_ 3.06).

The in vitro antidiabetic results using PTP1B and DPP4 targets are summarized in Table 2. In comparison to the alpha-glucosidase and alpha-amylase results, all of our compounds showed potentially active results against PTP1B and DPP4 enzyme targets. The observed and calculated IC_50_ values for compounds **10a**, **10b**, **10c**, **10d,** and **10e** were 11.99, 16.76, 18.98, 3.64, and 19.48 µM, respectively, in the PTP1B assay. Similarly, in the DPP4 assay, the observed IC_50_ values were 5.38, 20.51, 12.92, 4.22, and 14.67 µM for compounds **10a**, **10b**, **10c**, **10d,** and **10e,** respectively. So, overall, we observed that our compound **10d** was relatively more potent as compared to the other tested compounds. In both the enzymes’ activities, we observed that our compound **10d** was comparatively more potent than the respective standard drugs. In the PTP1B assay, the IC_50_ value of **10d** was observed as 3.64 µM in comparison to ursolic acid (a standard drug with an IC_50_ value of 11.98 µM). Similarly, in the case of DPP4, the observed IC_50_ of the compound **10d** was 4.22 µM in comparison to the IC_50_ of sitagliptin (IC_50_ 9.72 µM). In all the in vitro assays, we can easily conclude that the compound 10d with an N-benzyl substitution on the succinimide ring might have greater interactions with the tested enzymes’ targets. So, based on this overall potency, we selected only compound **10d** for onward in silico and in vivo studies.

### 2.3. Molecular Docking on Target Proteins

**Figure 3:**
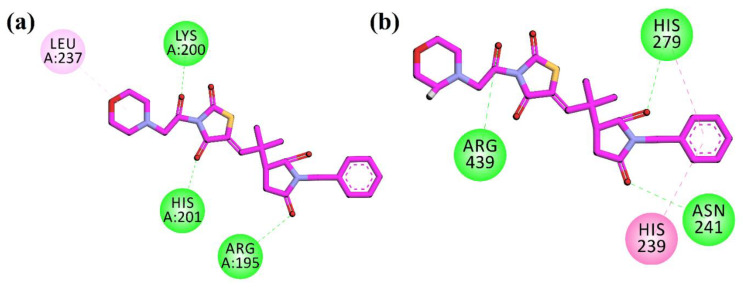
2D interaction plots of 10d in the binding sites of (**a**) α-amylase (4W93) and (**b**) α-glucosidase homology model.

To investigate the anti-diabetic potential of the synthesized compound **10d**, the three-dimensional structure of the compound was docked into the active site of the target protein using the docking software’s MOE (molecular operating environment). The crystal structures of α-amylase, α-glucosidase, DPP-4, and PTP-1B were retrieved from the protein data bank (PDB) using the four latter codes (PDB id), which are 4W93, and for the homology model, 1X70 and 1NNY, respectively. The dominant orientation was opted on the bases of the S-score and the interaction in the active site of the protein. The 2D plot of interaction was shown in Figure 3.

The compound was firstly docked into the active site of α-amylase (PDB ID = 4W93). The compound shows a hydrogen bonding interaction with the important residues of Arg195, Lys200, and His201, while Leu237 displayed a π-alkyl interaction in the active site of the target protein. In the active pocket of α-glucosidase (the homology model), the compound showed hydrogen bonding interactions with Asn241, His279, and Arg439. His239 showed π–π interactions with the phenyl ring. The 2D plot of the interactions of α-amylase and α-glucosidase are shown in Figure 3.

**Figure 4:**
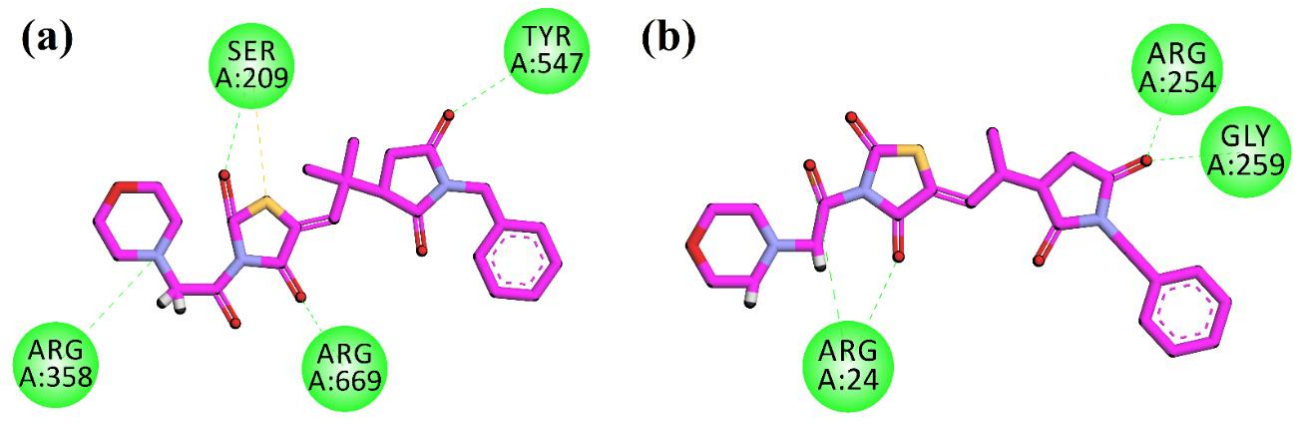
2D interaction plots of 10d in the binding sites of DPP-4 (1X70) (**a**) and PTP-1B (1NNY) (**b**).

The compound **10d** was docked into another anti-diabetic molecular target DPP-4 (PDB ID = 1X70). After the docking calculations and analysis, the resulting 2D plot of interaction is shown in Figure 4a. The compound showed hydrogen bonding interactions with Ser209, Arg358, Tyr547, and Arg669. Lastly, in the active pocket of PTP-1B (PDB ID = 1NNY), the compound show hydrogen bonding interactions with Arg4, Arg254, and Gly259 (Figure 4b). The molecular docking studies of the control compounds have been performed and shown in the supporting information Figures S1–4.

### 2.4. Acute Oral Toxicity Study

The limit test dose of 2000 mg/kg body weight was chosen for an acute toxicity study of the tested compound **10d** in experimental animal rats. In the LD_50_ evaluation, it was noticed that the experimental animals were safe and unharmed up to 2000 mg/kg body weight, because no abnormal changes in their behavior and no mortality was noticed up to this maximum dose. The LD_50_ of the compound **10d** as per the OECD guidelines falls under class IV values with no signs and symptoms of the acute toxicity at this maximum dose (2000 mg/kg). The pharmacological evaluations were carried out at doses of 10 and 20 mg/kg body weights.

### 2.5. In Vivo Antidiabetic Activity

**Figure 5:**
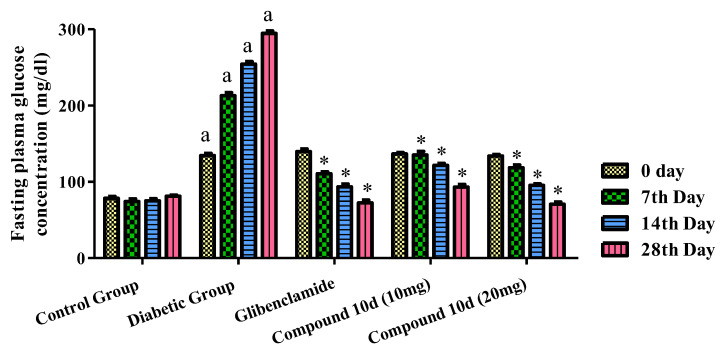
Effect of tested compound **10d** on fasting plasma glucose level. Values were presented as mean ± SEM (number of animals, *n* = 6) and were different significantly *^a^ p* < 0.05 in comparison to normal control group, * *p* < 0.05 when compared with diabetic control group.

The pharmacological evaluation for the antidiabetic activity of the tested compound **10d** was carried at the dose of 10 and 20 mg/kg by the method described previously. Glibenclamide was used as the standard. The different parameters used to check the antidiabetic activity was the fasting plasma glucose level, body weight, lipid profile, serum insulin level, and liver and renal serum biomarkers. The effect of the tested compound **10d** and glibenclamide on the levels of serum glucose in normal, tested, and diabetic rats is shown in Figure 5. In comparison to the normal experimental animals, there was a rise in the blood sugar level in the diseased rats. The STZ-induced rats were treated with two different concentrations of the compound 10d, i.e., 10 and 20 mg/kg b.w. We observed a significant fall in the blood glucose concentration to the normal range. The maximum level of the antidiabetic activity was achieved at the end of the 4th week (28th day). The observed blood glucose levels were 93.15 and 70.60 mg/dl at a concentration of 10 and 20 mg/kg of the compound **10d**. The glibenclamide-treated group displayed a 72.44 ± 3.58 mg/dl blood glucose level, respectively.

### 2.6. Effect of Tested Compound on Lipid Profile

**Figure 6:**
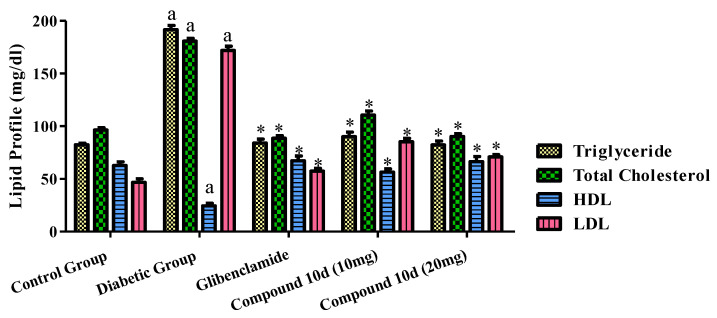
Effect of tested compound **10d** on lipid profile. Values were presented as mean ± SEM (number of animals, *n* = 6) and were significantly different at ^a^ *p* < 0.05 in comparison to normal control group, * *p* < 0.05 when compared with diabetic control group.

The effects of the tested compound and standard drug glibenclamide on the serum lipid profiles of the diabetic animals are displayed in Figure 6. The administration of glibenclamide and the test compound **10d** to the diabetic rats exhibited a significant decline in the total cholesterol level, triglyceride, LDL in the serum as compared to that of the diabetic control group. Although, the HDL level was greater than before in the groups treated with the tested compound **10d** and the standard drug evaluated to the diabetic control group.

### 2.7. Effect of Compound **10d** on Body Weight and Insulin Level

The diabetic control group’s animal body weight decreased significantly upon the induction of diabetes. The treated groups with the tested compound **10d** as well as glibenclamide were found to gain body weight in comparison to the diabetic control group, as displayed in Table 3. Similarly, a reduction in serum insulin level showed an increased glucose level in rats. On the 28th day of the study, STZ-induced diabetic rats displayed a significant decline in the serum insulin levels. The tested compound **10d** and glibenclamide administrations for the 28th day to diabetic rats resulted in a maximum increase in the levels of serum insulin.

### 2.8. Effect of Tested Compound 10d on Liver and Renal Serum Biomarkers

The elevated level of the ALT, AST, ALP, urea, LDH, and creatinine in the diabetic rat’s serum shows the abnormal function of the kidney and liver. The ALT, AST, ALP, urea, LDH, and Creatinine in the serum were significantly high in the diabetic control group in comparison to the normal control group. Similarly, the tested compound **10d** and the standard drug glibenclamide’s administration to the diabetic rats resulted in a significant reduction in the biochemical parameters, showing the improvement in the kidney and liver function (as shown in Table 4).

## 3. Discussion

Succinimide, chemically a pyrrolidinedione, has a close structural resemblance with the five-membered antidiabetic class of the compounds thiazolidinediones [32,33]. Pyrrolidinedione has been reported to have potential pharmacological activities [34,35,36,37]. Thiazolidinedione and its analogues have been proved to be potential antidiabetic compounds [25]. Similarly, previously, pyrrolidinedione has also been reported to possess a strong antidiabetic effect [38,39]. Moreover, chiral succinimides have already been reported to have a number of potential cardio and hepatoprotective activities [40]. Chiral compounds with absolute stereo purity have also been reported recently with a strong antidiabetic activity [41]. Morpholine analogue on thiazolidinedione has also been reported to have an antidiabetic potential [31]. In this research, we combined a chiral pyrrolidinedione (succinimide) moiety with thiazolidine derivatized with a morpholine unit. The multiple functionalities and double resemblance of the designed compounds mean that more potential antidiabetic compounds can be seen in the in vitro PTP1B and DPP4 results. As can be seen in Figure 1, we have obtained different *N*-substitution on our designed compounds (**10a**–**e**). All these compounds have possibly different interactions with the homology model of glucosidase and the target proteins of (from PDB) of alpha-amylase, PTP1B, and DPP4. The molecular docking studies (MOE) enable medicinal chemists to predict the biding mode of any compound with the minimized protein structure for the onset of different pharmacological activities [42,43,44]. Based on our in vitro results, we observed that the compound **10d** is a strong antidiabetic agent. Based on this scientific evidence, we docked the compound **10d** in the homology model (alpha-glucosidase) and protein structures (from PDB) of alpha-amylase, PTP1B, and DPP4. So, the compound **10d** with a benzyl substitution on the pyrrolidinedione ring with thiazolidinedione and morpholine moieties in its structure proved to be a new potential antidiabetic drug.

## 4. Materials and Methods

### 4.1. Step I: Reaction of Thiazolidinedione with Bromoacetyl bromide

Initially, the commercially available thiazolidine-2,4-dione (**1**, 1 equiv.) and K_2_CO_3_ (1 equiv.) were diluted in dimethylformamide (10 mL). Afterwards, the bromoacetyl bromide (**2**, 1.25 equiv.) was added dropwise. The reaction was setup and refluxed for 30 min at 120^o^ C. After the reaction, it was transferred to the cooled ice water for precipitation. The obtained precipitates were filtered out and washed three time with diethyl ether to obtain the desired product **3** [31].

### 4.2. Step II: Reaction of 3-(2-Bromoacetyl)thiazolidine-2,4-Dione with Morpholine

The previously synthesized compound **3** (1 equiv.) with morpholine (**4**, 1 equiv.) was dissolved in dimethylformamide (10 mL). Then, K_2_CO_3_ (1.5 equiv.) and a small amount of t-Bu-ammoniumbromide were added. The reaction was setup and refluxed for 30 min at 120 ^o^C. After completion, the solvent from the reaction was evaporated using a low-vacuum rotary evaporator. The reaction mixture was then diluted with water and ethyl acetate and the organic layer was extracted three times. Afterwards, the three organic (ethyl acetate) layers were combined and were concentrated using the same rotary evaporator. The final product **5** was crystallized as per the previous procedure [31].

### 4.3. Step III: Reaction of Isobutyraldehyde with Maleimides

In this organocatalytic asymmetric Michael addition, the commercially available isobutyraldehyde (**6**, 1.2 equiv.) was added with a catalytic amount of o-tert-butyl-L-threonine (**8**, 0.05 equiv.) and KOH (0.05 equiv.) in chloroform (1 mL). After a while of stirring, N-substituted maleimide (**7**, 1 equiv.) was added and the Michael addition reaction continued. The reaction was complete in 3 h. After completion, the reaction was transferred to a separating funnel with the additional chloroform (10 mL). Water (10 mL) was added to the diluted reaction and the organic (chloroform) layer was separated. The same was repeated three times. The three layers were combined and then concentrated. The product was obtained as pure product **9** and confirmed by ^1^H NMR [45].

### 4.4. Step IV: Synthesis of Succinimide–Thiazolidinedione Derivatives

The compound **5** obtained in step II was reacted with the Michael product **9** obtained in Step III in a equimolar amount in ethanol. The reaction was catalyzed with a small amount of pyridine. The reaction progressed was checked for 24 h. After 24 h, the compound **10** was purified by precipitation and crystallization. The structure of the compound **10** was determined with ^1^H and ^13^C NMR analyses [39].

### 4.5. α-Glucosidase Inhibitory Activity

Using Homo sapiens α-glucosidase, the in vitro inhibitory potentials of all of the aforementioned synthesized succinimides and thiazolidinedione hybrids **10a–e** were investigated. The inhibitory analyses for α-glucosidase were carried out in accordance with the protocols reported in the literature; the inhibitory activities of the synthesized hybrids were calculated utilizing screening assay tools [31]. The efficiencies of the synthesized hybrids were determined by calculating the quantity sufficient to inhibit 50% of the enzyme (IC_50_ in M) and the values of the enzymes’ inhibitions of synthesized hybrids are shown in molar concentrations. For the in vitro enzyme inhibition analysis, the marketed medication acarbose was chosen as a standard drug and the synthesized compounds’ in vitro findings were compared to the reference drug. The IC_50_ values show that the majority of our compounds suppressed α-glucosidase, mostly with half maximal inhibitory concentration (IC_50_) values varying in the range [46].

### 4.6. In Vitro α-Amylase Inhibitory Activity

The inhibiting effectiveness of the synthesized compounds **10a**–**e** on the amylase enzyme was investigated. The samples of the compounds **10a–e** in five different concentrations ranged from 500 to 31.25 μM. The test samples were added in alpha-amylase solution. The enzyme solution was prepared with enzyme and buffer solution. The mixture of the samples and enzyme was then incubated. Then, starch and dinitrosalicylic acid solution was added. The same was performed for both test compounds and the standard drug. The sample mixture was heated for a short while and then the absorbance was measured at 656 nm as per the reported procedure. The percent alpha-amylase inhibition activity and its IC_50_ values were calculated and confirmed as per the previous procedure [31].

### 4.7. PTP1B Assay

The aforementioned newly synthesize succinimides and thiazolidinedione hybrids were tested against the PTP1B enzyme to determine their ability to inhibit the negative regulation of insulin as well as leptin signaling. The PTP1B test was performed with a small modification to the method described in the publications. The enzyme efficiency was evaluated using p-nitrophenyl phosphatase (pNPP) as a substrate. For the buffer solution, 0.5 millimolar (mM) of ethylene diamine tetraacetic acid (EDTA), 1 mM of mercaptoethanol, 0.55 mM of dithiothreitol (DTT), and 12.49 mM of TRIS hydrochloride (ph = 7.4) were used. The experiment was carried out by mixing 10.0 μL of the test compound solutions with 20.0 μL of the enzyme (1 μg/mL), subsequently injecting 40.0 μL of 4.0 mM 4-nitrophenyl dihydrogen phosphate in 130.5 μL of the prescribed buffer in a microplate well at 37 °C for 10 min. pNP was formed during the enzymatic process, which was monitored for 30 min. The IC_50_ values were derived using a non-linear regression analysis of the fraction of the product generated vs. the log of the inhibitory concentration [41].

### 4.8. DPP-4 Activity Bioassay

All succinimides and thiazolidinedione hybrids were tested for DPP-4 inhibitory effects on a human DPP-4 enzyme using the methodology published before [4]. The DPP-4 kit was used to measure the inhibition. L-thiazolidine (P32/98) was included as a reference. The hybrids and DPP-4 enzyme were both adulterated 1/50 in the assay buffer (PH = 7.4, 25 mM Tris). Following that, a microplate well was filled with 5 μL of assay buffer, 3 μL of enzyme solution, and 2 μL of adequately diluted test chemical solutions. After a 12-min incubation period at 37.5 °C, a diluted substrate solution of 25 μL was added into the solution. A multi-mode reader was used to assess the fluorescence. GRAPHPAD PRISM5 was used to calculate the IC_50_ values and compare the inhibition ratio to the control without the inhibitor. Overall, the hybrids are more effective than the reference compound [41].

### 4.9. Molecular Docking

The docking study were performed to explore the anti-diabetic inhibition potential of the compound **10d** using molecular operating environment (MOE) software [47]. The 3D structure of the compound **10d** was docked into the active site of the diabetic target α-amylase, α-glucosidase, DPP-4, and PTP-1B. the crystal structures of the target proteins were obtained from the protein data bank (PDB) using the four latter accession code (PDB id) [48]. The PDB id for α-amylase is 4W93, for α-glucosidase (the homology model) the DPP-4 is 1X70, and for PTP-1B is 1NNY. Following the previously reported standard procedure, the docking compound was first protonated and then minimized. The redocking strategy was used to conform molecular docking. Upon completion, the best orientation was selected on the bases of the S-score and an interaction in the active site and comparison of the native ligand with the respective enzyme. The 2D interactions were analyzed using Discovery Studio Visualizer (DSV-2020) software.

### 4.10. Animals

Inbred Wister Albino rats weighing 150–230 gm were utilized for our studies. In the antidiabetic studies, rats of either sex weighing 150–230 gm were used, whereas for the toxicity studies, rats aged 8–12 weeks were used. The animals were acclimatized for a period of seven days to the laboratory environment before all the experimental procedures and were given sufficient food, water, and ad libitum.

### 4.11. Toxicological Screening

For the acute toxicity study, a limit test on the laboratory animals at 2000 mg/kg body weight was selected and the up and down procedure (UDP) was followed as per the guidelines of the Organization for Economic Cooperation and Development (OECD). The purpose was to evaluate the dose necessary to perform various pharmacological activities.

### 4.12. Selection of animals

Healthy young adult male rats of about 8 to 12 weeks old were selected for dosing.

### 4.13. Preparation of Animals

The rats were randomly selected, marked for an individual identification, and retained in their cages for at least 5 days before dosing to acclimatize to the laboratory conditions. Prior to dosing, the animals were fasted and they were refrained from food, not water, for 16–18 h [9].

### 4.14. Calculation and Administration of Dose

After the fasting period, the weight of each animal was determined and according to the body weight of the animals, the dose at 2000 mg/kg body weight was calculated for each animal. A unique dose of the studied compound **10d** was administered to the animals for the UDP study by the oral route using an intragastric cannula 16 G, and it was planned to dose the five animals on the same day. After the 1^st^ 30 min of dosing, the animals were observed at least once; after the first 24 h, the animals were observed periodically with special care given during the first four hours and thereafter observed daily for 14 days. The onset time, toxic reactions, and length of the recovery period determined the duration of the observation and when considered necessary, may be extended. The important duration is the time at which toxicity signs appear and disappear. Individual observations were recorded and maintained systematically for each animal. Observations such as changes in the mucous membranes and eyes, skin, and fur, the circulatory, respiratory, central, and autonomic nervous systems, the somatomotor activity, and the behavior pattern were included. Observations of the salivation, tremors, convulsions, lethargy, diarrhea, sleep, and coma were also considered [18].

### 4.15. Antidiabetic Activity

The antidiabetic activity of the synthesized compound **10d** was evaluated using a streptozotocin-induced diabetic rats model.

### 4.16. Animals Selection, Care, and Handling

Male Wistar rats weighing 150 to 230 gm were used for the study which were housed in polyacrylic cages (no more than six animals per cage) with dimensions of 38 × 23 × 10 cm and maintained under standard laboratory conditions with a dark/light cycle of 14 h of dark and 10 h of light at a temperature of 25 °C. The animals were provided with a standard diet (dry pellet) and water ad libitum. Before the start of the experimental procedures, the rats were acclimatized to laboratory conditions for 10 days. All the experiments were approved and performed as per the guidelines of the Departmental Research Ethics Committee under letter No. DREC/050 [49].

### 4.17. Induction of Non-Insulin-Dependent Diabetes Mellitus (NIDDM)

Non-insulin-dependent diabetes mellitus was induced in fasted rats weighing 150–230 gm using a single dose of streptozotocin (STZ) at a dose of 60 mg/kg intraperitoneally after 15 min of the administration of nicotinamide at a 120 mg/kg dose i.p. Nicotinamide was prepared in normal saline while in citrate buffer (pH 4.5), STZ was prepared. After the induction of diabetes, the increase in the plasma glucose level was measured at 72 h and then measured on the 7th day to confirm hyperglycemia [49]. For the diagnosis of diabetes, the threshold value of the fasting plasma glucose level was considered to be more than 126 mg/dl. For the study, only the animals with permanent NIIDM were utilized.

### 4.18. Experimental Design

The animals were classified into 5 groups with 6 animals in each group (*n* = 6). Group I comprised of normal control rats which were daily administered drinking water only; group II served as diabetic control rats (disease group); group III served as the positive control and were administered 0.5 mg/kg of the standard drug glibenclamide; and group IV and V served as the test group rats and were administered the test compound **10d** at 10 mg/kg and 20 mg/kg, respectively, for twenty-eight days. The fasting glucose concentration was measured on the 0, 7th, 14th, and 28th day of the administration of the extract. During the experimental tenure, the weights of the rats were determined daily and the mean change was recorded [18].

### 4.19. Insulin Level Estimation

After the 28th day of treatment with the test compound **10d,** the blood samples were taken for the determination of the insulin levels, which were measured with the help of GLAZYME INSULIN-EIA TEST.

### 4.20. Determination of Biochemical Parameters

The animals were euthanized (sacrificed) via cervical dislocation on the 28th day to observe the biochemical parameters. The parameters studied were triglyceride (TGL), cholesterol, low-density lipoprotein, and high-density lipoprotein (LDL and HDL). The Minias Globe Diagnostic kits were used for cholesterol and TGL. Semi-autoanalyzers were used for the analysis. The serum samples from the rats were also checked for alanine aminotransferase (ALT), aspartate aminotransferase (AST), lactate dehydrogenase (LDH), serum alkaline phosphatase (ALP), and creatinine and urea levels utilizing commercially available kits of Crest Biosystems [50]. The statistical analysis was also performed as per the previous procedure [51].

## 5. Conclusions

Based on our overall results in this research paper, we can conclude that we have synthesized and explored a new class of compounds as potential antidiabetic agents. In the in vitro experiments against α-glucosidase, α-amylase, PTP1B, and DPP4 targets, our compound **10d** was observed to be the most potent, exhibiting the lowest IC_50_ values. Using the MOE, we also performed the molecular docking studies of the compound **10d** against the target enzymes α-glucosidase, α-amylase, PTP1B, and DPP4, which shows overwhelming interactions. Based on the in vitro and in silico studies, we subjected the compound **10d** to the in vivo experiments using experimental animals, which overall gave us encouraging results. The type of compound (**10d**) with a benzyl substitution can be further derivatized for the enhanced activity. Moreover, the antidiabetic activity of these compounds can also be explored of further receptors and human organs.

## Figures and Tables

**Table 1:** In vitro α-glucosidase and α-amylase antidiabetic potential of test compounds **10a–e**.

Compound	Conc (µM)	% α-Glucosidase Inhibition	IC_50_ (µM)	% α-Amylase Inhibition	IC_50_ (µM)
**10a**	500 250 125 62.5 31.25	79.49 ± 0.6 *** 75.58 ± 0.63 *** 72.93 ± 0.67 ** 65.44 ± 0.58 *** 54.56 ± 1.73 ***	16.5	82.61 ± 0.77 *** 77.60 ± 0.80 *** 72.83 ± 0.56 ** 57.55 ± 0.77 ^***^ 54.58 ± 0.74 ***	21.9
**10b**	500 250 125 62.5 31.25	79.48 ± 0.74 *** 75.62 ± 0.40 *** 70.60 ± 0.46 *** 57.68 ± 0.49 *** 51.72 ± 0.66 ***	27.4	79.37 ± 0.69 *** 74.72 ± 0.51 *** 70.47 ± 0.59 *** 65.50 ± 0.71 *** 48.46 ± 0.72 ***	21.20
**10c**	500 250 125 62.5 31.25	81.53 ± 0.71 *** 76.58 ± 1.12 *** 71.42 ± 0.43 *** 65.08 ± 0.47 *** 62.72 ± 1.01 ***	25.6	88.58 ± 1.12 ** 81.65 ± 1.34 ** 74.31 ± 2.15 ** 67.56 ± 1.73 *** 62.44 ± 0.58 ***	28.3
**10d**	500 250 125 62.5 31.25	88.43 ± 1.26 *** 83.83 ± 0.66 ^ns^ 77.93 ± 0.90 ^ns^ 72.26 ± 0.77 * 67.10 ± 0.95 **	10	82.33 ± 1.20 *** 76.33 ± 0.95 *** 72.67 ± 0.91 *** 70.00 ± 0.17 ** 68.60 ± 0.04 ^ns^	12
**10e**	500 250 125 62.5 31.25	86.44 ± 0.58 ** 81.08 ± 0.47 * 79.84 ± 0.30 ^ns^ 74.94 ± 1.13 ^ns^ 52.58 ± 0.63 ***	13.2	92.67 ± 1.30 ^ns^ 88.58 ± 0.47 ^ns^ 82.54 ± 0.68 ^ns^ 78.20 ± 1.24 ^ns^ 73.40 ± 0.42 ^ns^	9.66
**Acarbose**	500 250 125 62.5 31.25	94.40 ± 0.03 85.03. ± 2.16 80.90 ± 1.11 76.44 ± 0.28 71.22 ± 0.47	6.80	93.08 ± 1.04 86.45 ± 0.90 80.58 ± 0.63 75.40 ± 0.20 70.80 ± 0.90	3.06

Data were analyzed as mean ± SEM, two-way ANNOVA pursued by Bonferroni test was followed for the statistical analysis. *** *p* < 0.001, ** *p* < 0.01, * *p* < 0.05, ns: not significant (*p* value less than 0.05).

**Table 2:** In vitro PTP1B and DPP4 antidiabetic activities of compounds **10a**–**e**.

Compound	Conc (µM)	PTP1B	IC_50_ (µM)	DPP4	IC_50_ (µM)
**10a**	500 250 125 62.5 31.25	88.61 ± 0.43 *** 82.58 ± 0.63 *** 75.10 ± 0.60 *** 69.25 ± 1.40 *** 62.87 ± 0.85 ***	11.99	87.45 ± 0.59 ** 82.49 ± 0.60*** 77.23 ± 0.44 *** 72.50 ± 0.61 *** 67.47 ± 0.46 ***	5.38
**10b**	500 250 125 62.5 31.25	85.32 ± 2.87 *** 77.12 ± 0.54 *** 72.79 ± 1.08 *** 65.79 ± 1.88*** 57.20 ± 0.47 ***	16.76	81.30 ± 1.42 ** 75.78 ± 0.45 ** 69.44 ± 0.86 * 62.72 ± 1.89 ** 54.29 ± 2.64 **	20.51
**10c**	500 250 125 62.5 31.25	79.37 ± 1.04 *** 73.37 ± 0.54 *** 69.30 ± 2.61 *** 63.42 ± 1.05 *** 53.52 ± 2.52 ***	18.98	77.40 ± 1.51 *** 72.57 ± 3.84 *** 67.36 ± 0.55 *** 62.56 ± 0.95 *** 57.37 ± 1.10 ***	12.92
**10d**	500 250 125 62.5 31.25	89.88 ± 0.89 ^ns^ 84.54 ± 3.60 *** 79.01 ± 1.97 *** 74.68 ± 0.22 *** 71.82 ± 1.95 ***	3.64	91.36 ± 1.49 ^ns^ 85.34 ± 0.55 ^ns^ 81.39 ± 2.49 ** 76.47 ± 0.52 *** 71.44 ± 2.55 ***	4.22
**10e**	500 250 125 62.5 31.25	83.51 ± 0.54 ** 75.76 ± 1.61 *** 67.22 ± 1.28 *** 63.51 ± 0.54 *** 56.37 ± 2.56 ***	19.48	88.52 ± 2.06 ** 82.48 ± 0.60 ** 74.54 ± 1.46 ** 67.34 ± 2.63 *** 61.30 ± 1.49 ***	14.67
**Ursolic acid**	500 250 125 62.5 31.25	94.08 ± 1.04 87.45 ± 0.90 81.58 ± 2.63 76.40 ± 3.20 71.80 ± 0.90	11.98	-	-
**Sitagliptin**	500 250 125 62.5 31.25	-	-	97.23 ± 0.82 92.45 ± 0.90 85.90 ± 0.60 81.00 ± 3.30 76.90 ± 1.45	9.72

Table: the effect of tested compounds. The values were represented as mean ± SEM. * *p* < 0.05, ** *p* < 0.01, *** *p* < 0.001 and ns not significant compared to standard drugs. Data were analyzed via two-way ANOVA pursued by the Bonferroni test.

**Table 3:** Effect of compound **10d** on body weight and insulin level.

Group	Change in Body Weight (gm)			Insulin Level (Mean ±SEM) in mg/dl	
	Before Induction	After Induction	After Treatment	Initial Reading	Final Reading
**Control Group**	190.13 ± 1.52	192.24 ± 1.45	188.35 ± 3.65	0.76 ± 0.12	0.80 ± 0.75
**Diabetic**	188.30 ± 2.10 ^ns^	172.80 ± 1.63 ^###^	146.68 ± 2.78 ^###^	0.84 ± 0.26 ^ns^	0.26 ± 0.56 ^ns^
**Glibenclamide**	180.66 ± 1.67 ^ns^	160.52 ± 2.23 **	176.90 ± 4.45 ***	0.82 ± 0.34 ^ns^	0.80 ± 0.16 ^ns^
**Comp 4 (10mg)**	191.23 ± 1.50 ^ns^	154.22 ± 3.62 ***	182.43 ± 5.76 ***	0.81 ± 0.25 ^ns^	0.65 ± 0.57 ^ns^
**Comp 4 (20mg)**	204.66 ± 3.60 ***	171.60 ± 1.90 ^ns^	198.19 ± 4.66 ***	0.76 ± 0.38 ^ns^	0.76 ± 0.81 ^ns^

Values are presented as mean ± SEM (number of animals, n = 6) and were significantly different at ^###^ *p* < 0.001 in comparison to normal control group, while *** *p* < 0.001, ** *p* < 0.01, ns *= p* > 0.05 when compared with diabetic control group.

**Table 4:** Effect of tested compound **10d** on liver and renal serum biomarkers.

Treatment Group	AST (U/L)	ALT (U/L)	ALP (U/L)	LDH (U/L)	Urea (mg/dl)	Creatinine (mg/dl)
**Control Group**	96.24 ± 2.53	52.66 ± 3.25	145.43 ± 1.73	852.33 ± 5.83	48.22 ± 2.44	0.34 ± 0.12
**Diabetic**	555.52 ± 4.80 ^###^	366.70 ± 4.58 ^###^	612.35 ± 4.43 ^###^	1536.43 ± 6.90 ^###^	122.56 ± 3.46 ^###^	0.80 ± 0.03 ^ns^
**Glibenclamide**	80.36 ± 2.12 ***	76.23 ± 2.13 ***	124.42 ± 2.35 ***	826.21 ± 5.60 ***	42.45 ± 2.21 ***	0.44 ± 0.04 ^ns^
**10d (10mg)**	123.40 ± 3.70 ***	136.80 ± 2.15 ***	184.43 ± 3.83 ***	930.11 ± 4.26 ***	75.26 ± 2.44 ***	0.51 ± 0.57 ^ns^
**10d (20mg)**	94.32 ± 2.30 ***	60.42 ± 2.20 ***	122.80 ± 2.00 ***	850.53 ± 4.41 ***	51.53 ± 2.23 ***	0.42 ± 0.31 ^ns^

Values were presented as mean ± SEM (number of animals, *n* = 6) and were significantly different at ^###^ *p* < 0.001 in comparison to normal control group, while *** *p* < 0.001 when compared with diabetic control group, ns (not significant); *p >* 0.05.

## Data Availability

The whole data are available within the manuscript and the supporting information.

## Supplementary Materials

The following are available online at https://www.mdpi.com/article/10.3390/molecules28031207/s1.

## Sample Availability

The samples are available from the authors.
