# Supplementary material for: New Succinimide–Thiazolidinedione Hybrids as Multitarget Antidiabetic Agents: Design, Synthesis, Bioevaluation, and Molecular Modelling Studies
Source: Molecules. 2023 Jan 26;28(3):1207. doi: 10.3390/molecules28031207 (PMC9918900; doi:10.3390/molecules28031207)
Supplement: Supplementary file 1 [file molecules-28-01207-s001.zip › molecules-2139781 - Supporting Information.pdf]

molecules

Supporting Information

New succinimide-thiazolidinedione hybrids as multitarget antidiabetic agents: Design, synthesis, bioevaluation and molecular modelling studies

Mohammed A. Huneif [1], Mater H. Mahnashi [2,*], Muhammad Saeed Jan [3], Muhammad Shah [4], Sultan A. Almedhesh [1], Seham M. Alqahtani [1], Mohammad Jamaan Alzahrani [1], Muhammad Ayaz [5], Farhat Ullah [5], Umer Rashid [4,*] and Abdul Sadiq [5,*]

1 Pediatric Department, Medical College, Najran University, Najran 55461, Saudi Arabia
2 Department of Pharmaceutical Chemistry, College of Pharmacy, Najran University, Najran 55461, Saudi Arabia
3 Department of Pharmacy, Bacha Khan University, Charsadda 24461, Pakistan
4 Department of Chemistry, COMSATS University Islamabad, Abbottabad Campus, Abbottabad 22060, Pakistan
5 Department of Pharmacy, Faculty of Biological Sciences, University of Malakand, Chakdara 18000, Pakistan
* Correspondence: matermaha@gmail.com (M.H.M.); umerrashid@cuiatd.edu.pk (U.R.); sadiquom@yahoo.com (A.S.); Tel.: +966-508734539 (M.H.M.); +92-(0)301-2297-102 (A.S.)

Citation: Huneif, M.A.; Mahnashi, M.H.; Jan, M.S.; Shah, M.; Almedhesh, S.A.; Alqahtani, S.M.; Alzahrani, M.J.; Ayaz, M.; Ullah, F.; Rashid, U.; et al. New Succinimide–Thiazolidinedione Hybrids as Multitarget Antidiabetic Agents: Design, Synthesis, Bioevaluation, and Molecular Modelling Studies. *Molecules* **2023**, *28*, 1207. https://doi.org/10.3390/molecules28031207

Academic Editors: Aleksandra Misicka-Kesik

Received: 19 December 2022
Revised: 18 January 2023
Accepted: 22 January 2023
Published: date

Copyright: © 2023 by the authors. Submitted for possible open access publication under the terms and conditions of the Creative Commons Attribution (CC BY) license (https://creativecommons.org/licenses/by/4.0/).

Binding Orientations of Control

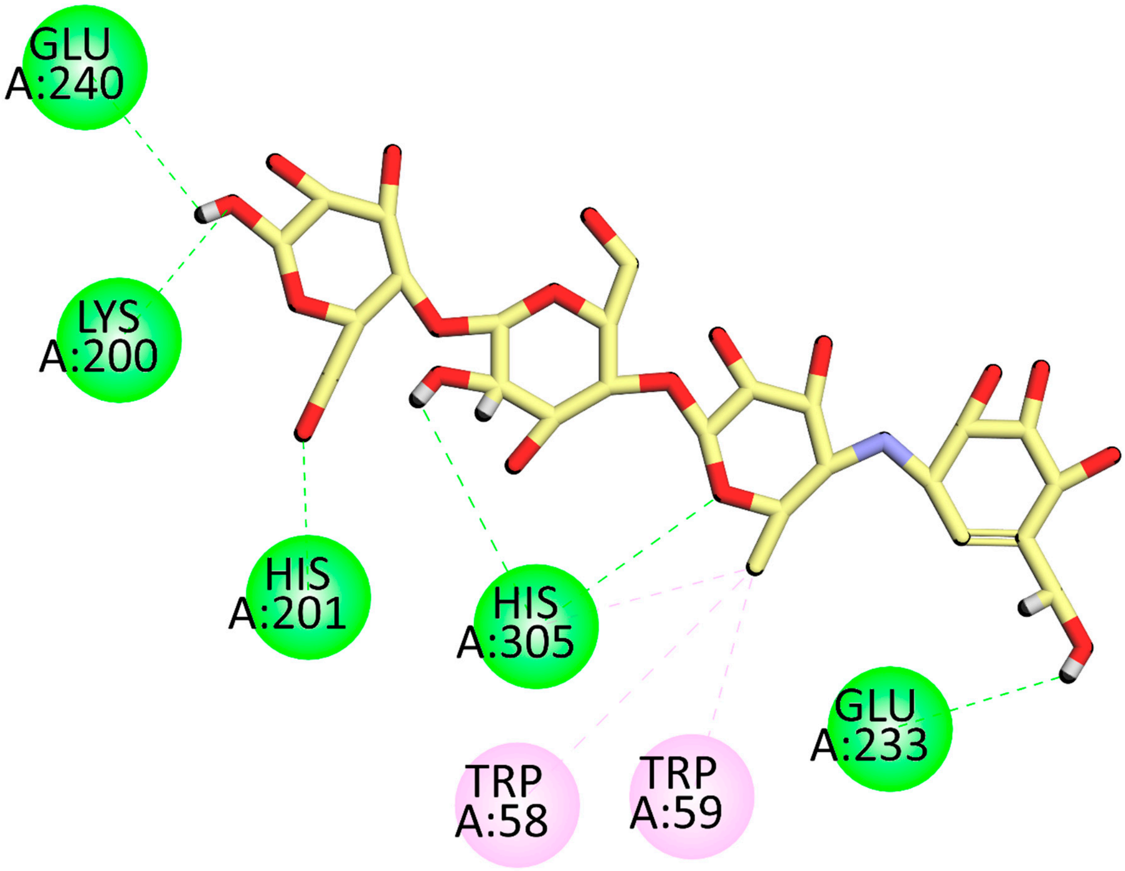

Figure S1: 2D interaction plot of acarbose in the binding site of α-Amylase.

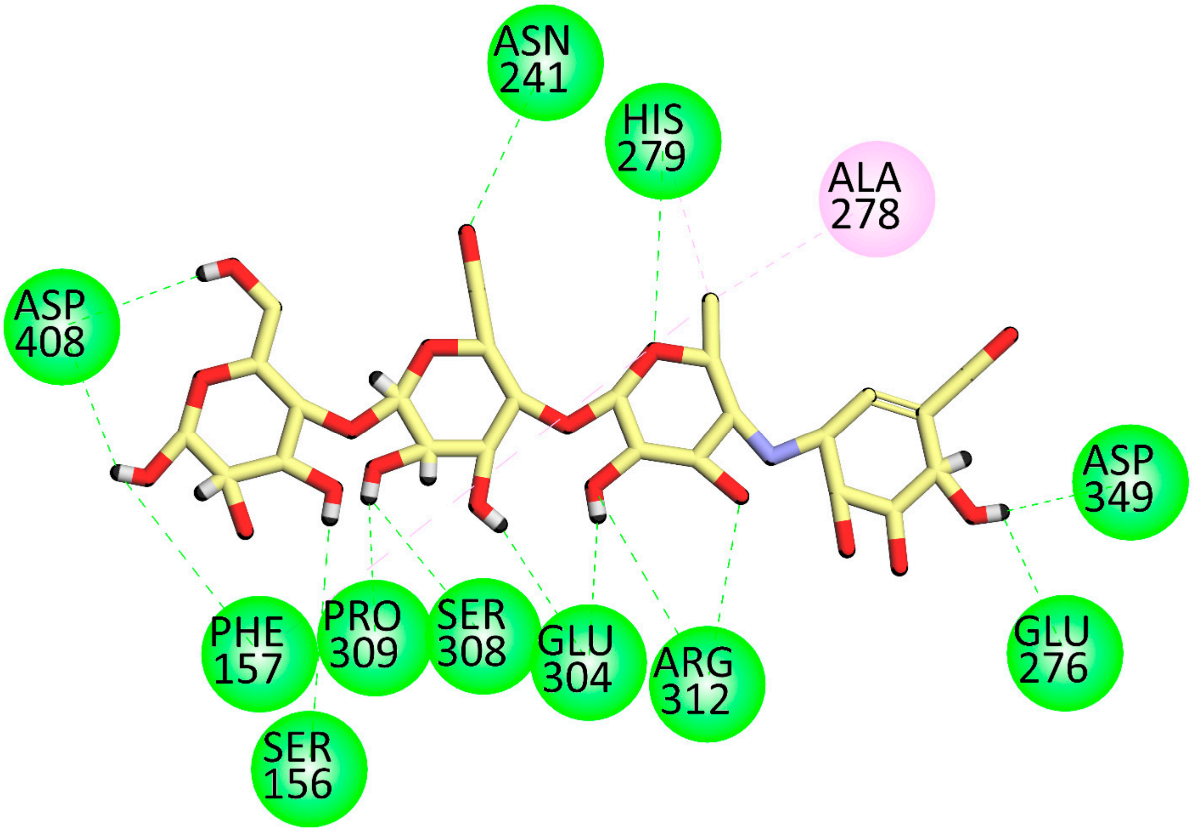

Figure S2: 2D interaction plot of acarbose in the binding site of α-Glucosidase.

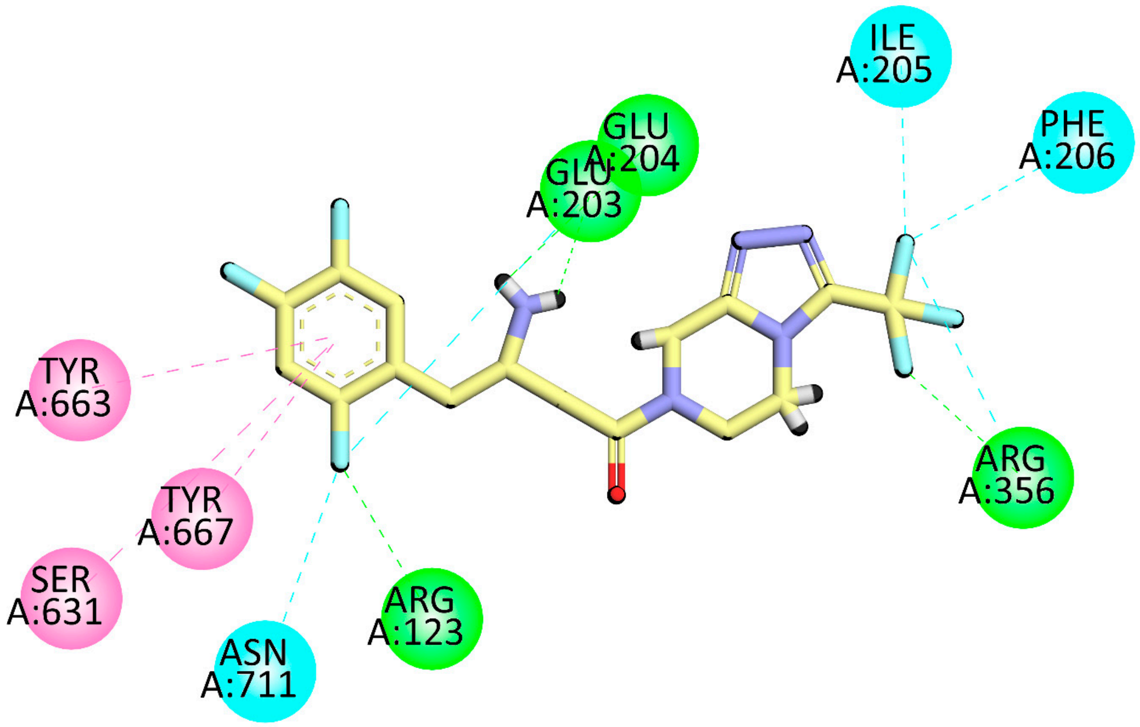

Figure S3: 2D interaction plot of sitagliptin in the binding site of DPP4.

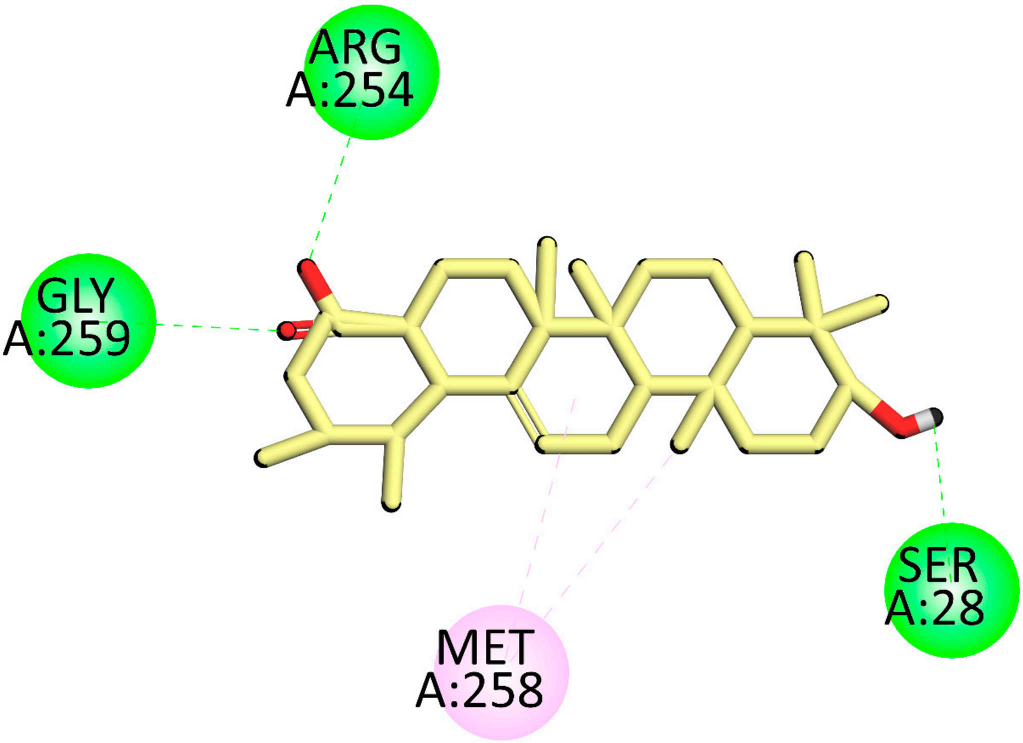

Figure S4: 2D interaction plot of ursolic acid in the binding site of PTP-1B.
